# Hybrid Epoxy Composites with Both Powder and Fiber Filler: A Review of Mechanical and Thermomechanical Properties

**DOI:** 10.3390/ma13081802

**Published:** 2020-04-11

**Authors:** Danuta Matykiewicz

**Affiliations:** Institute of Materials Technology, Faculty of Mechanical Engineering, Poznan University of Technology, Piotrowo 3, 61-138 Poznań, Poland; danuta.matykiewicz@put.poznan.pl

**Keywords:** hybrid epoxy composites, glass, carbon, basalt, fiber, powder filler, mechanical and thermomechanical properties

## Abstract

Fiber-reinforced epoxy composites are used in various branches of industry because of their favorable strength and thermal properties, resistance to chemical and atmospheric conditions, as well as low specific gravity. This review discusses the mechanical and thermomechanical properties of hybrid epoxy composites that were reinforced with glass, carbon, and basalt fabric modified with powder filler. The modification of the epoxy matrix mainly leads to an improvement in its adhesion to the layers of reinforcing fibers in the form of laminate fabrics. Some commonly used epoxy matrix modifiers in powder form include carbon nanotubes, graphene, nanoclay, silica, and natural fillers. Fiber fabric reinforcement can be unidirectional, multidirectional, biaxial, or have plain, twill, and satin weave, etc. Commonly used methods of laminating epoxy composites are hand lay-up process, resin transfer molding, vacuum-assisted resin transfer molding, and hot or cold pressing. The following review is a valuable source of information on multiscale epoxy composites due to the multitude of technological and material solutions.

## 1. Introduction

Polymer hybrid materials contain fillers or modifiers that have different functionalities, thanks to which they are characterized by unique usable, technological, and processing properties. Designing a composite material requires taking the processing technique, the susceptibility of all components to heat, mechanical treatment, durability, and the ability to create compounds into account [1,2,3,4,5]. Epoxy resins can be in the form of liquids, pastes, or powders, which significantly facilitates their modification by mixing with reactive or non-reactive additives [6,7,8]. Epoxies, due to a wide range of hardeners, can cure at room or elevated temperature, achieving favorable mechanical and thermal properties. However, they are reinforced with synthetic and natural fibers, such as glass, carbon [9], basalt [10], aramid [11], jute [12], or flax [13,14], due to their low impact strength and brittleness. Hybrid epoxy composites may contain several types of powder or liquid modifiers, several types of fibrous modifiers, or both powder and fibrous modifiers [15,16]. A specific type of a composite for chemo- and thermosetting resins is laminates, in which the reinforcing component is placed in layers and connected with the polymer matrix [17,18]. The properties of laminates are particularly influenced by: the number of layers used, the type and direction of reinforcement fibers, the properties of the matrix used, the production method, and the adhesion between all of the components of a composite [19,20]. The combined reinforcement layers forming the composite structure exhibit better mechanical properties in the plane, but are much weaker in the transverse direction. Therefore, when designing laminates, one of the limiting factors may be the poor strength between layers. In response to this phenomenon, scientists modify the properties of the resin, the surface of the fibers, or apply inter-layer sewing. Subsequently, they analyze the types of damage that may appear in composites such as interlaminar, intralaminar and translaminar, of which interlaminar is most common [20]. In the case of the number of layers used in the composite, it was proved that the mechanical behavior of the laminated material was improved by increasing the number of layers [21]. Placing fabrics in a laminate at different angles (0°, ±45°, 90°) reduces their anisotropic characteristics. Favorable mechanical and dynamic properties were observed for materials reinforced with fabric at an angle 0 and 45 degree [22]. The production process of laminates has a special impact on their properties and, above all, the appropriate supersaturation of all layers of resin fibers in order to obtain a finished product. Improper methods of manual lamination, infusion, or vacuum bags often cause structural defects in the material that reduce its mechanical strength and impact strength. In addition, many epoxy resins require heating at high temperatures to fully cure, and due to the different thermal expansion properties of the fiber and matrix, after cooling, there might be residual thermal stress in the laminates [19]. In order to produce a layered composite with the given properties, the properties of all of its components should be taken into account with respect to the manufacturing method.

Numerous studies focus on the assessment of the impact on the characteristics of the epoxy materials of powder additives, such as: carbon nanotubes (CNTs), graphene; graphite, graphene, graphene nanosheets, fullerenes [23], metal carbides [24], silica [25], hydrated alumina powder [26], or natural filler [27,28]. Additionally, a lot of studies describe the modification of epoxy resin by simultaneously introducing two or more types of powder fillers into the epoxy matrix. For example, Zhou et al. [29] applied micro- and nano-fillers, such as micro-SiC and multi-walled carbon nanotubes (MWCNTs), to increase the thermal conductivity of the composites. The epoxy matrix with 6 wt.% MWCNTs or 71.7 wt.% micro-SiC showed 2.9 and 20.7 times higher the thermal conductivity than the unmodified epoxy resin, respectively. In addition, the application of the both MWCNT and micro-SiC fillers increase the thermal conductance 24.3 times when compared to pure epoxy polymer. The hybrid filler consisted of 5 wt.% silane-treated carboxyl-functionalized MWCNTs + 55 wt.% oxidized and silane-treated micro-SiC. Tang et al. [30] examined the influence of the incorporation of spherical particles, such as nano-silica or submicron-rubber and carbon nanotubes, on the electrical and mechanical characteristics of epoxy polymer. These high-performance ternary hybrid composites were characterized by a favorable combination of such properties as glass transition temperature values, electrical conductivity, stiffness, and strength, as well as fracture toughness. Qing et al. synergistically investigated reinforced epoxy composites via nitrogen-doped graphene and titanium carbide nanosheet. Their results showed that these are highly promising fillers for such materials as thin-thickness, broadband absorption microwave absorbers [31]. Likewise, Polydoropoulou et al. described the synergistic effect of the introduction of MWCNTs and glycidyl polyhedral oligomeric silsesquioxanes (GPOSS) to the properties of thermoset resin [32]. For epoxy materials with the addition of both MWCNTs and GPOSS, the compressive and flexural strength and G_IC_ fracture toughness decreased when compared to the reference sample. The authors attributed this effect to the MWCNTs agglomerates and the GPOSS aggregates present in the epoxy resin. Qi et al. also described a synergistic effect on the enhancement of mechanical features of composites after introducing graphene oxide and carbon nanotubes into liquid resin [33]. 

In addition to different types of powder modifiers, the epoxy matrix is often reinforced using many types of fibers, such as glass fiber (GF), carbon fiber (CF) [34], basalt fiber (BF) [35], and natural fiber [36,37,38]. The blending of fibers improves the mechanical and thermal stability of epoxy composites modified with natural fibers, as reported by Mittal et al. [39]. Therefore, applying two or more types of reinforcement in the form of fibers or fabrics is a popular method of preparing hybrid epoxy materials. Dutra et al. presented the result of the hybridization of polypropylene (PP) or mercapto-modified polypropylene blend fibers (PPEVASH) on the impact resistance and dynamic mechanical characteristics of carbon fiber reinforced epoxy composites [40]. The hybridization of CF/epoxy materials with PP or PPEVASH significantly improved the impact resistance of the composite.

The impact properties under low impact velocity of glass/basalt woven fabric reinforced epoxy hybrids were reported in work [41]. Basalt and hybrid composites (basalt-skin/glass-core) showed a higher impact energy absorption capacity and damage tolerance ability than glass laminates. However, the most beneficial flexural properties were exhibited by composites with symmetrical sandwich-like configuration (basalt-skin/glass-core type). The results presented by Ramesh et al. proved that sisal/glass fiber epoxy laminates possess better tensile properties than jute/glass fiber epoxy laminates, which, however, showed better flexural properties than sisal/glass reinforced composites [42]. Dong and Davis examined the flexural and tensile moduli for GF/CF reinforced hybrid epoxy materials in intra-ply configurations [43]. As the modulus of GF is much less than that of CF, both flexural and tensile modulus were reduced with the growing hybrid ratio. The carbon/epoxy composite flexural modulus was lower when the CF on the compressive side was substituted by GF [43]. When more glass fibers were introduced, the flexural modulus remained stable. For full glass/epoxy composites, the flexural modulus was low. Gupta and Rao investigated sisal/hemp fiber epoxy hybrid composites [44], which were introduced at concentrations of 10, 20, 30, 40, and 50 wt.%. The flexural strength of the laminate improved with fiber concentration up to 40 wt.%. Potluri et al. described epoxy composites that were reinforced with okra/kenaf and okra/banana fibers [45]. Introducing banana fiber into okra fiber/epoxy composites improved their tensile properties. James et al. assessed the mechanical and morphological characteristics of epoxy composites with sisal/bagasse fibers [46]. They concluded that laminates with three sheets of sisal fibers in the center exhibited favorable features. Tripathi et al. considered the influence of alkali treatment, soil degradation, and water absorption on the strength of jute-bagasse-glass/epoxy-based hybrid composite [47]. The tensile strength and hardness of natural fiber hybrid materials depended on the glass fiber content, and increased with weight concentration. On the other hand, in paper [48], two sandwich-structured hybrid composite materials were fabricated from Aloe vera fiber, ceramic fiber wool, glass fiber with the epoxy polymer matrix and from sisal fiber, ceramic fiber wool, glass fiber with epoxy polymer matrix. The mechanical analysis confirmed that the sisal fiber reinforced laminates had better mechanical properties than the Aloe vera fiber reinforced laminates.

The main purpose of this article is to describe epoxy hybrid composites that contain both powder and fiber filler in their structure. The modification of the epoxy matrix by particles and simultaneous reinforcement with fibers allows for obtaining the unique properties of such composite materials.

## 2. Hybrid Epoxy Composites Reinforced with both Powder and Fibrous Filler 

### 2.1. Hybrid Epoxy Composites Reinforced with Glass Fiber

Glass fiber-reinforced plastics, in particular epoxy glass-reinforced composites, are used in the construction, aviation, and maritime industries due to their advantageous mechanical properties, lightness, resistance to atmospheric conditions, and low price. That is why they remain the subject of numerous studies into fiber arrangement and modification of the epoxy matrix through nano- and micro-additives, despite other types of reinforcing fibers appearing on the market [49,50].

Böger et al. studied the fatigue properties of the epoxy laminates containing two different types of E-glass fibre non-crimp-fabrics and modified with fumed silica SiO_2_ and multi-walled carbon nanotubes at a low concentration (0.3 wt.%) [51]. For the modified composites rises in load cycles by orders of magnitude were observed in tensile, alternating, and compression loading in both cases (with fumed silica and MWCNT). These results may be attributed to the higher inter fiber fracture strength of the composites. Rahman et al. manufactured epoxy composites that were reinforced with E-glass woven fabric modified with amino-functionalized MWCNT (0.1–0.4 wt.%) with enhanced mechanical and thermomechanical properties [52]. For the composites containing 0.3 wt.% MWCNTs, maximum enhancements in strength by 37%, in modulus by 21%, in strain to failure by 21%, in storage modulus by 41%, in loss modulus by 52%, and an increase of 10°C in the glass transition temperature in relation to reference samples were observed.

The thermal properties of epoxy laminates that were reinforced with E-glass fabric and modified by graphene nanoplatelets (GNPs) at 2 wt.% depending on the post-curing time and temperature were evaluated by Seretis et al. [53]. Different relations between mechanical and thermal features were observed. The best mechanical properties of composites were obtained for such post-curing parameters as low temperature in long time and higher temperature and short time. For most post-cured materials (75%), the loss of weight began later than for original materials. Likewise, Moriche et al. manufactured a multiscale epoxy composite reinforced with glass fabric and modified by 12 wt.% GNPs [54]. No improvement in mechanical properties was observed for these composites due to the weak interface between the glass fiber and the doped epoxy resin, which enhanced the detrimental effect of the accumulation bands of this GNPs. Furthermore, Tuncer and Canyurt evaluated the influence of the addition of graphene nanoparticle (0.1%, 0.3%, and 0.5% by weight) on the tensile strength of E-glass/epoxy composite [55]. The authors designed two types of composites with different fiber placement type A (0°/+45°/−45°/90°/0°/90°/−45°/+45°/0°), type B (90°/+45°/−45°/0°/90°/0°/−45°/+45°/90°). The matrix properties were enhanced by the introduction of the graphene nanoparticle; the composites with f 0.1% graphene additive showed 20% growth in the type A composite and 22% in type B composite strength.

Additionally, the application of epoxy with the addition of GNPs, CNTs, hexagonal boron nitride nanosheets (BNNS), and boron nitride nanotubes (BNNT) nanoparticles as the matrix for the composites reinforced with glass fiber fabric was described in another study [56]. The authors assessed the ballistic impact behavior and damage mechanisms of the glass fiber reinforced plastics (GFRP) modified with different nanoparticles (0.25 wt.% GNP; 0.1 wt.% CNT; 0.1 wt.% CNT:0.1 wt.% BNNS and 0.25 wt.% GNP: 0.1 wt.% BNNT. The effects of full-field deformation, exit velocity, and energy absorption studies from the ballistic examinations exhibited significant enhancements in impact resistance for the nanomodified composites epoxies in comparison to the pure epoxy based materials. The highest absolute absorbed energy was indicated for the GFRP materials that were produced from the epoxy resin with BNNT/GNP.

Zeng et al. studied the influence of graphene oxide (GO) dimensions on interlaminar shear property of glass fabric/epoxy (GF/epoxy) structural materials [57]. The results proved that at a low GO concentration (≤0.1 wt.%), the interlaminar shear strength (ILSS) of the GF/epoxy composites increased with the GO size. Hence, the ILSS of the composites first increased, and then decreased with the increasing GO concentration. This effect might be caused by GO agglomeration. Introducing the large (129.6 μm) GO at 0.05 wt.% content resulted in the greatest enhancement in the ILSS, with a 83.7% increase.

The effect of montmorillonite (MMT) clay particles (1% and 3% nanoclay doping ratios) on the incubation period in solid particle erosion of glass fibers fabric/epoxy nanocomposites was presented in work [58]. The unmodified glass fiber/epoxy composite showed the highest erosion resistance in comparison to the modified composites. This effect resulted from the agglomeration and weak compatibility of nanoclay, glass fiber, and epoxy matrix. Furthermore, the montmorillonite smectite clays particles (1; 3; 5 and 7 wt.%) were applied to epoxy composites that were modified with woven fabric glass fiber [59]. For composites with 5 wt.% clay, the tensile strength increased by 23.58% and modulus by 23.66%, in comparison to the unfilled composite. Additionally, for the composites with 5% the flexural strength modulus and impact strength increased by 34.10% and 53.86%, respectively, in reference to the unmodified sample. A higher addition of nanoclay resulted in a decrease in the tensile and flexural properties of the composite. The impact strength value increased with an increase in nanoclay up to 3 wt.%. Additionally, Withers et al. reported the improved mechanical property which resulted from the addition of surface organo modified nanoclay (quaternary ammonium salt bentonite) at concentration of 2 and 4 wt.% to epoxy composites reinforced with fabric of E-glass fibers [60]. The monotonic tensile tests (60 °C/air) of the modified composites exhibited an average enhancement in the ultimate tensile strength (by 11.7%), tensile modulus (by 10.6%), and tensile ductility (by 10.5%) in comparison to the reference sample. In tension–tension fatigue examinations (stress proportion +0.9; 60 °C/air), the composite with nanoclay indicated 7.9% higher fatigue strength and a fatigue life over a decade longer than the unmodified composites.

**Figure 1:**
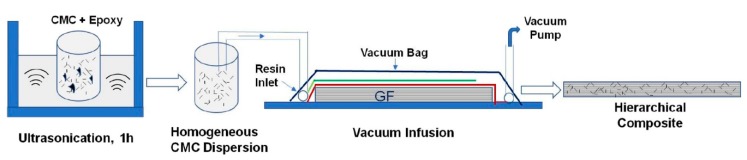
Production scheme for hierarchical materials [61]. (From open access publication).

**Figure 2:**
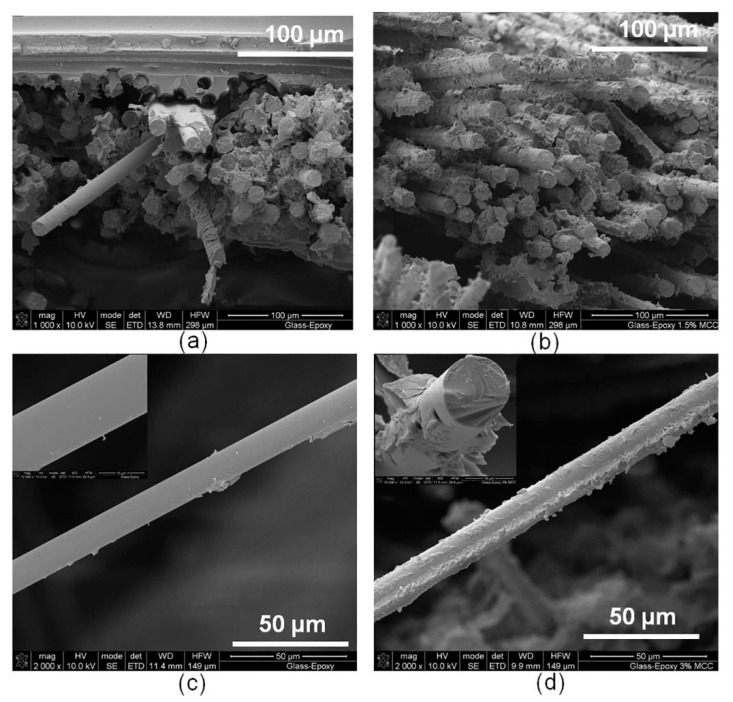
Surface of: glass/epoxy materials (**a**), composites materials with cellulose microcrystals (CMC) (**b**); structure of single fiber glass in: unmodified glass/epoxy materials (**c**) and composites materials with CMC (**d**) [61]. (From open access publication).

Cellulose microcrystals (CMCs) at concentration of 1–3 wt.% were successfully used to improve the fiber-matrix interface, mechanical, dynamic mechanical, and thermal behavior of epoxy laminates reinforced with glass woven fabric [61]. Figure 1 presents the production scheme for these hierarchical materials. The composites with higher CMC concentrations possessed agglomerates. Therefore, the composites with 1 wt.% CMCs were characterized by the best properties, such as a 65% improvement in ILSS, 14% and 76% improvement in tensile and flexural strengths, respectively, improvements in the fracture energy in tensile test (111%) and in flexural test (119%), and a 9.4% enhancement in the impact resistance. Moreover, 13.5% higher storage modulus values, 21% loss modulus, and 13 °C glass transition temperature increase were observed for the modified materials. Figure 2 shows the fracture surface of investigated materials. 

Naidu et al. evaluated the mechanical properties of epoxy hybrid composites that were reinforced with woven glass fiber and modified with the graphitic carbon nitride (g-C3N4) at concentrations of 1%, 1.5%, 2%, 2.5%, and 3% [62]. The best properties were found for composites with 2% of g-C3N4; the tensile strength increased by 11% and the flexural strength increased by 13%. The dispersion between the matrix and the modifier was uniform at concentration from 0 to 2% filler and the ILSS strength increased steadily. For a filler concentration above 2%, the particles were agglomerating in composites and the mechanical properties decreased. In turn, glass/epoxy composites that were modified with ammonium polyphosphate (APP) and melamine polyphosphate (PNA) at concentrations of 5, 10, and 20 wt.% and manufactured by the resin powder molding process were studied in [63]. Especially for samples containing 5 wt.%, the tensile strength and thermal stability of the hybrids increased, like in APP and PNA. Moreover, the limiting oxygen index (LOI) of the modified composites was enhanced following the addition of ammonium polyphosphate and melamine polyphosphate, from 26 LOI to 32 LOI, and better flame resistance indicated samples with PNA.

Shah et al. applied different bamboo powder (BP) concentrations to modify the glass fiber-reinforced epoxy hybrid composites with a sandwich-structure from the top layer, and the bottom layer from woven E-glass fiber [64]. The composite with a low BP addition of 10% showed the highest tensile strength. An opposite effect was noticed in flexural strength; the maximum value was observed for composites with the highest BP addition of 30%. Rajasekar et al. [65] used natural bio additives, such as coconut shell (CS) and tamarind seed (TS) particulates, to modify GF/epoxy laminates with proportion of 100 fiber to one filler, and assessed their the fracture toughness. Glass/epoxy laminates without the filler exhibited the best properties. The maximum critical load with crack initiation of GFRP/epoxy composites with bio fillers decreased accordingly: GF/epoxy laminate + 1% CS, GF/epoxy laminate + 0.5% CS + 0.5% TS, and GF/epoxy laminate + 1% TS. 

Based on the above examples, it can be stated that the main use of hand lay-up and compression hot press techniques allowed for producing stable fiber glass reinforced laminate structures. In order to obtain a hybrid structure, the epoxy matrix was modified by introducing powder fillers into it and then using it in the manufacturing process. When introducing a nano or micro powder filler into the epoxy system, its concentration should be selected in order to enable the modified resin to be produced in a specific production process. Table 1 presents the characteristics of glass fiber reinforced hybrid composites. 

By introducing carbon materials in nanometric sizes, the highest efficiency for hybrid composites was obtained while using modifier concentrations in the range of 0.1–0.5 wt.%. When used as MWCNT modifier, both the mechanical high-speed mixer and sonication methods allowed for obtaining a homogeneous suspension for the lamination process. Favorable mechanical properties were obtained by introducing MWCNT in a concentration of up to 0.3 wt.%. For epoxy laminates reinforced with glass fabric and modified graphene nanoplates, improved mechanical properties (mainly tensile strength and ballistic impact) were obtained by introducing this modifier into the resin at 0.1 wt.%. Additionally, the interlaminar shear strength composites increased when graphene oxide at low concentration (≤0.1 wt.%) is introduced into the epoxy matrix. As the modifier’s particle size increases, its higher concentrations (1–5 wt.%) should be used to improve the desired properties. Explicit enhancement in the tensile strength and flexural strength was obtained after the introduction of MMT into the epoxy matrix. Cellulose microcrystals have also proved to be an effective modifier in relation to the epoxy polymer, enabling the improvement of the mechanical properties of laminates that were made using the vacuum bag method. Techniques for producing fiber glass-reinforced epoxy composites are well developed. However, the methods of improving the adhesion between the fiber and matrix and the mechanical properties of the composite through the introduction of powder fillers each time require the development of a special technology for mixing components, methods for applying them to fibers, and a curing scheme for epoxy materials.

### 2.2. Hybrid Epoxy Composites Reinforced with Carbon Fiber

Carbon fibers are infusible high strength and stiffness materials made of graphitic and non-crystalline regions. They are chemically inert, electrically conductive, and have a relatively low coefficient of thermal expansion [66]. That is why they are widely used as reinforcing materials in lightweight polymer composite materials, mainly for the construction parts of aircraft, spacecraft, boats, or cars [67,68].

**Figure 3:**
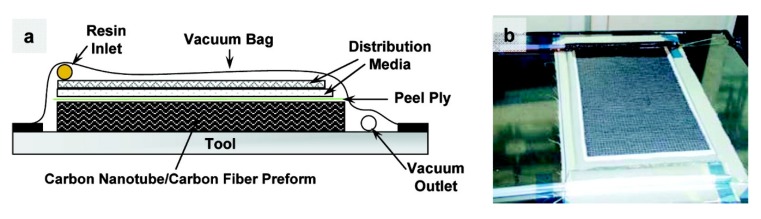
Scheme (**a**) and picture (**b**) showing the infiltration of the CNT/carbon fiber performs by vacuum-assisted resin transfer molding [73]. (Reprinted with permission from Langmuir 2007, 23, 7, 3970-3974. Copyright 2007 American Chemical Society).

**Figure 4:**
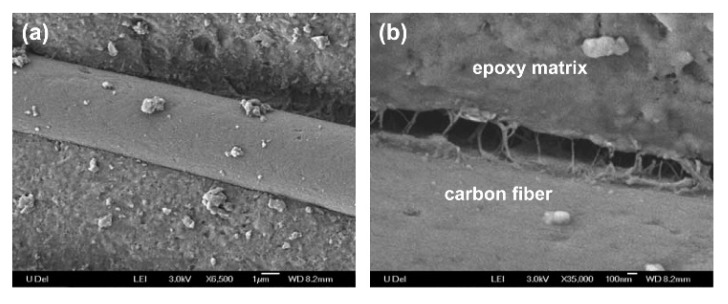
SEM micrographs of a modified CF into the composite: (**a**) with low enlargement at 1 µm; (**b**) with high enlargement at 100 nm [73]. (Reprinted with permission from Langmuir 2007, 23, 7, 3970-3974. Copyright 2007 American Chemical Society).

Jiang et al. proposed an interesting method of epoxy matrix modification through introducing graphene oxide sheets onto carbon fiber surface. The team managed to significantly improve the interfacial shear strength of the CF-GO/epoxy composite in comparison to composites without GO [69]. Yao et al. [70] used the CNT-coated, GO-coated, and virgin uni-directional carbon fiber fabrics for CF/epoxy composites. The addition of both CNTs and GO notably improved the interfacial properties of the laminates. The epoxy structural materials with CNT_S_ showed higher shear strength, and the epoxy structural materials with GO showed higher humidity resistance. CNT coating improved the surface roughness of the carbon fiber and increased the contact area between the fiber and the matrix. On the other hand, the GO coating interacted with resin and created interfacial mechanical interlocking and chemical bonding. The synergistic effect of modifying the carbon fabric-reinforced epoxy composites with layered graphene nanoplatelets and multi walled carbon nanotubes was described in work [71]. In all cases, nano-fillers increased the interlaminar fracture toughness of the composites when compared to the reference material. Mode I fracture toughness (G_IC_) was the highest for 0.5 wt.% GNPs + 1 wt.% MWCNTs addition (45% higher than the reference sample). The same hybrid composites indicated the highest value of (G_IIC_) Mode II fracture toughness (25% higher than the reference sample). Likewise, Sánchez et al. presented the interesting results of their study into carbon fiber composites with modified epoxy resin matrices via amino-functionalized and non-functionalized carbon nanotubes [72]. These hybrid composites, produced via vacuum assisted resin infusion molding (VARIM), had improved mechanical performance, such as flexural strength, as wells as both intra and interlaminar strengths, especially for composites filled with functionalized CNTs. For these materials the nanoreinforcement effect and matrix strengthening were observed. In another study two types of carbon nanotubes MWNTs and single-walled carbon nanotubes (SWNTs) were applied to deposition on a carbon fabric area by electrophoresis [73]. The carbon nanotube-carbon fabrics were infiltrated by epoxy using vacuum-assisted resin transfer molding (VARTM). Figure 3 presents a diagram showing the composites manufacturing process. The nanoscale reinforcement strongly affected the mechanical properties and electrical conductivity of the fiber composites. The strong bonding between the fibers and matrix was proven by SEM analysis (Figure 4). The addition of 0.25% of MWNTs into epoxy composites reinforced with CF improved the ILSS by 27%. The tensile strength and tensile modulus values of the MWNT-modified composites were similar to the unmodified composites. Moreover, the MWNT/CF/epoxy laminates indicated an improvement of the out-of-plane electrical conductivity of 30% in comparison to the original sample.

Composites that were based on epoxy resin reinforced with carbon fiber, polyhedral oligomeric silsesquioxane (POSS), and CNT were also presented in work [74]. Grafting CNTs on the carbon fiber area via octaglycidyldimethylsilyl POSS as coupling agent improved the interfacial interaction between CF and the epoxy resin. Moreover, the roughness and polar functional groups of the CF increased following the modification. Consequently, the impact resistance, ILSS, and dynamic mechanical property of the composites were enhanced. Additionally, ordered multi walled carbon nanotubes were used to improve the micro-cracks resistance of the CF/epoxy laminate [75]. The ordered Fe_3_O_4_/MWCNTs showed enhanced mechanical properties, such as tensile strength, the impact strength of epoxy matrix at 77 K, as well as micro-cracks resistance, and a lower coefficient of thermal expansion (decreased by 37.6%) of the epoxy matrix.

Dorigato et al. [76] applied epoxy-clay nanocomposites (organically modified montmorillonite with loading of 5 wt.%) as a matrix for epoxy-reinforced continuous carbon fibers composites. The tensile properties of composites slightly increased in comparison to the pristine epoxy composites. Moreover, a significant improvement in the energy absorption capability of the modified composites was observed. Hosur et al. used epoxy with organically modified montmorillonite nanoclay to fabricate carbon/epoxy composite laminates [77]. The introduction of MMT into the epoxy matrix lowered the impact damage and improved the stiffness and resistance to damage progression of the nanophased composites. Peng et al. prepared carbon fabric epoxy composites containing organically modified montmorillonite with greater mechanical properties [78]. Modified MMT was obtained by ion-exchanging natural MMT with the hydrochlorate of triglycidyl para-aminophenol (TGPAP), a tertiary amine-type epoxy oligomer applied as a composites matrix in the aerospace industry. The incorporation of TGPAP-modified MMTs delayed the propagation of inter-layer delamination, and during the three-point bending tests it was observed that fiber breakage was the main reason for damage. The introduction of 4 wt.% of TGPAP-modified MMTs increased the ILSS of the carbon fiber/epoxy composites by 52% and the flexural strength by 52.3%.

Guo et al. [79] prepared composites that were filled with hybrid nano-SiO_2_ particles and short carbon fibers. Moreover, nano-SiO_2_ particles were grafted with copolymer of styrene and maleic anhydride. The synergetic effect of both fibrous and powder filler in hybrid composites was observed, together with improved surface hardness and tribological performance. On the other hand, Landowski et al. [80] used industrial surface-modified nanosilica (1–8 wt.%) to modify epoxy resin as a matrix for composites reinforced with carbon fiber fabric. They assessed impact parameters: force, deformation, energy, and damage dimension. The most favorable properties were found for composites with 8% nano-SiO_2_ content. Moreover, a decrease in permanent deformation by ~15% and absorbed energy by ~8%, as well as better fiber/matrix interfacial strength were observed. In other studies, prepregs of carbon fibers with and without surface-modified silica nanoparticle (SiO_2_) were used to prepare a laminate via compression molding [81]. The laminate that was modified by silanized silica showed better ILSS by 45% and impact toughness by 32%, flexural strength by 32%, and flexural modulus by 50% than the laminate with untreated fibers. Moreover, a review of use of silica nanoparticles in GF/CF epoxy composites was presented in work [82]. The authors exhibited that the introduction of silica nanoparticles to GF-epoxy nanocomposite or CF-epoxy nanocomposite enhanced some mechanical properties, such as tensile strength, fracture toughness, impact and fatigue properties. Alsaadi et al. conducted an interesting study into the results of nano-silica inclusion (at concentration of 0.5, 1, 1.5, 2.5, and 3 wt.%) on the mechanical and dynamic properties of carbon/Kevlar fiber fabric-reinforced epoxy resin hybrids [83]. The addition of nano-silica particles (NS) to these composites improved both tensile and flexural strength. The authors attributed this effect to the excellent adhesion of NS particles with epoxy/fiber composites, which resulted in an increase in the load transfer between the particle and the matrix. The 20% enhancement in tensile properties was noted for the laminates with 3 wt.% NS, while the flexural strength increased by 35.7% in comparison to the virgin material. 

In turn, the hybridization of the carbon fiber-reinforced epoxy composites by nano- and micro-sized Al_2_O_3_, fillers (10 vol.%) improved the mechanical properties of these materials [84]. Unidirectional carbon fibers were pulled through the resin bath with the filler, winded on mandrel, cut into flat sheets, and then pressed into samples. A higher flexural and interlaminar shear strength was observed for the samples with the Al_2_O_3_ filler.

Bunea et al. studied the effect of epoxy resin features, amount of carbon and aramid sheets, and fiber direction on low velocity impact properties of the structural hybrid composites [85]. The epoxy matrix was filled with potatoes starch, carbon black, aramid powder, barium ferrite, carbon, and glass whiskers (at 20% total volume ratios). Moreover, the researchers investigated the effect of the number carbon and aramid sheets in laminates and fiber orientation (at 0°, ±15°, ±30°, ±45° angles) on their impact properties. Hybrid composites with 0° ply orientation showed the best impact resistance value. The matrix properties had a significant influence over the fracture mode of the hybrid composites; the fault degree of the damaged areas was closely associated with fiber orientation. 

Carbon fiber reinforced laminates exhibit high specific stiffness and strength in various structural applications. Therefore, the method of modification of these materials cannot lead to a deterioration of their original properties. Table 2 presents characteristics of carbon fiber reinforced hybrid composites. As in the case of hybrid composites with glass fiber, the concentration of introduced modifiers was closely related to their structure and size. Carbon derivatives, such as: CNTs, GNPs, and GO introduced into the epoxy matrix in a concentration of up to 1 wt.% provided enhancement of the mechanical properties of laminates. For this group of hybrid materials, vacuum-assisted production methods ensured the proper connection of all components. An interesting effect of adding MMTs to the epoxy matrix was the propagation delay of inter-layer delamination of carbon fiber reinforced composites. In turn, the introduction of nano-SiO_2_ particles into the epoxy system in an amount of up to 8 wt.%, allows for improving the interfacial strength, impact toughness, and flexural strength of layered materials.

### 2.3. Hybrid Epoxy Composites Reinforced with Basalt Fiber

Basalt fiber is obtained through molting basalt rock and fiber spinning, which is a more eco-friendly process than producing other fibers, such as glass and carbon. Basalt is made of plagioclase, pyroxene, and olivine. Its fibers display high strength and modulus in comparison to glass fiber and, moreover, they are chemically stable, non-toxic, and non-combustible [86]. Therefore, they successfully replace carbon fiber in composite materials with unique properties [87].

Chen et al. manufactured and investigated cross-ply composites reinforced with basalt fibers and functionalized MWCNTs at concentration of 0.5 vol.% or 1.5 vol.% from unidirectional epoxy prepregs [88]. The distribution of MWCNTs into the epoxy matrix was improved by their surface modification and, as a result, the mechanical properties of the composite were enhanced. Study [89] presented the consequence of seawater absorption on the damping and fracture properties of the epoxy/basalt fiber composites (basalt/CNT/epoxy) modified with 1 wt.% silanized carbon nanotubes. The vibration damping coefficient of basalt/CNT/epoxy laminates increased by approximately 50% by seawater absorption. The average fracture toughness of the composites affected by seawater was approximately 20% lower in comparison to original samples, as swelling of the epoxy matrix decreases interfacial bonding between the components in this material. Furthermore, Lee et al. evaluated the impact of the addition of CNTs on the tensile and thermal properties of CNT/basalt/epoxy materials [90]. Woven basalt fibers were impregnated by epoxy resin that was mixed with unmodified, oxidized, and silanized CNTs at a concentration of 1 wt.%. The silanized CNT/basalt/epoxy composites indicated improved tensile properties in comparison to the unmodified and oxidized CNT/basalt/epoxy composites. When compared to the unmodified composites, the tensile strength of the silanized composites was 34% higher, and the Young’s modulus was 60% higher. Moreover, silanized materials indicated better thermal stability, storage modulus, and glass transition value than unmodified and oxidized composites. Additionally, Kim et al. used silanized and oxidized carbon nanotubes to modify epoxy/basalt hybrids [91]. The flexural behaviors of epoxy/basalt composites were enhanced by the introduction of CNTs. The silanization process of the CNTs improved the interfacial bonding between the epoxy resin and basalt fibers, enabling effective stress transfer in the structure of the composite. The flexural modulus of epoxy composites with silane-treated CNT was 54% higher and flexural strength was 34% higher when compared to unmodified epoxy composites. 

**Figure 5:**
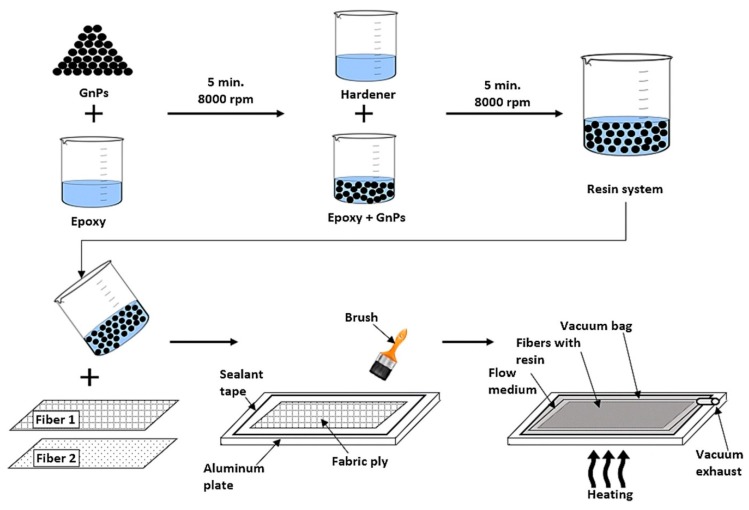
Manufacture method of the nanographene/basalt fiber composite plate [93]. (Reprinted with permission from J. Brazilian Soc. Mech. Sci. Eng. 2020, 42, 83. Copyright 2020 Springer Nature).

**Figure 6:**
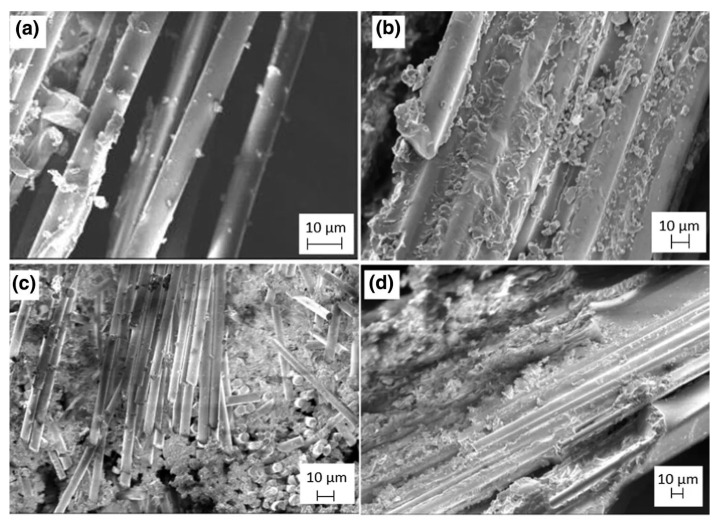
SEM micrograph of (**a**) glass/epoxy composites; (**b**) glass/epoxy composites with 0.1 wt.% GNP; (**c**) glass/basalt/epoxy composites; and, (**d**) glass/basalt/epoxy composites with 0.1 wt.% GNP [93]. (Reprinted with permission from J. Brazilian Soc. Mech. Sci. Eng. 2020, 42, 83. Copyright 2020 Springer Nature).

Study [92] assessed the mechanical characteristics of basalt/epoxy laminates that were modified with graphene nanopellets at concentrations of 0.1, 0.2, and 0.3 wt.%. For composites that were modified with 0.1 wt.% GNPs, the highest mechanical values, such as tensile strength, flexural strength, and impact resistance were obtained. Likewise, Erkliğ and Doğan introduced nanographene to hybrid epoxy composites (0.1; 0.25; 0.5 wt.%) reinforced with woven S-glass fabrics and basalt fabric [93]. Figure 5 presents the manufacturing method of the composites. Both composite types with basalt and glass at outer and inner skins were analyzed in impact and mechanical examination.. The introduction of GNP at 0.1 wt% resulted in significantly increased impact and mechanical properties of the epoxy composites. In addition, in the case of hybrids with PNB, rough surfaces of GF and BF fiber were observed, which significantly improves the adhesion of the fiber to the polymer matrix in such materials (Figure 6). Similarly, Kazemi-Khasragh et al. analyzed the influence of the addition of surface-modified graphene nanopellets at concentrations of 0.1, 0.2, 0.3, 0.4, and 0.5 wt.% on the sliding wear behaviors and microhardness of woven basalt fibers/epoxy composites made by the hand lay-up method [94]. The introduction of surface-modified GNPs into the epoxy matrix enhanced the microhardness and wear properties of these composites. Composites with 0.5 wt.% GNP obtained the best microhardness result (21.7 HV). The highest wear properties were obtained with 0.3 wt.% GNPs with loads of 20 and 40 N. The GNPs agglomerated and wear properties decreased above 0.3 wt.%. Interestingly, Kim used natural graphite flakes (NGF) (10, 20, 30, 40 wt.%.) to fabricate basalt woven fiber-reinforced epoxy composite by the hand lay-up and pressing method [95]. Graphite flakes notably improved the mechanical properties of the composites, especially the critical stress intensity factor (K_IC_) and critical strain energy release rate (G_IC_). Composites with 20 wt.% of NGF showed the best mechanical properties and thermal stability. Thermal conductivity increased with the NGF concentration in the composites.

Jamali et al. [96] studied the influence of silane-treated (as N-(3-trimethoxysilylpropyl)ethylenediamine) graphene oxide nanoplatelets (0.1; 0.2; 0.3; 0.4; 0.5 wt.%) on the mechanical properties of basalt fiber/epoxy composites. Composites with 0.4 wt.% silanized GO (SGO) exhibited the maximum improvement by 18% in tensile strength, 59% in the flexural strength, and 61% in the compressive strength out of the investigated group of materials. For the sample with 0.5 wt.% silanized GO, the maximum improvement by 46%, 54%, and 66% in the tensile, flexural, and compressive modulus was noted, respectively. The SGO addition resulted in a higher mechanical property enhancement than the GO addition. In another study [97], the viscoelastic and tribological properties of these materials were investigated. For the basalt/epoxy composites with 0.4 wt.% SGO enhanced tribological properties, such as wear rate (62% lower than the reference sample) and friction coefficient (44% lower than the reference sample), were observed. Moreover, the composites with 0.4 wt.% SGO showed the increase of 130% in the storage modulus and of 13.6 °C in the glass transition temperature in comparison to the unmodified sample. Toorchi et al. assessed the effect of the addition of nano-zirconium oxide (ZO) (1; 2; 3 wt.%), graphene oxide (0.1; 0.3; 0.5 wt.%), and ZO + GO on the basalt/epoxy laminate [98]. Nano-zirconium and graphene oxide were treated with 3-aminopropyltrimethoxysilane. The composites with 2 wt.% ZO + 0.1 wt.% GO showed the best values of energy absorption (increased by 67%), impact limit velocity (increased by 30%), and ILSS (increased by 77%). The analysis of the fracture surface of the studied materials confirmed that the presence of nanofillers in the epoxy resin enhanced the adhesion between the BF and epoxy polymer.

Moreover, halloysite nanotube (HNT) at concentrations of 1.0, 2.0, and 3.0 wt.% was applied to modify the epoxy resin matrix for basalt fiber reinforced composites [99]. These hybrid laminates were subjected to aging in a salty water environment and their tensile, flexural, and dynamic-mechanical properties were investigated. Generally, the introduction of HNTs into epoxy resin significantly improved the mechanical properties of the basalt-reinforced composites. The composites with 2 wt.% HNT showed the best mechanical properties. The authors attributed this effect to toughening mechanisms such as: crack deflection, crack pinning and bowing related to the one-dimensional (1D) morphology of halloysite nanotube. Long-term salty water exposure reduced the tensile strength, flexural strength and fracture toughness of the composites due to the weakening of the fiber-matrix connections. The composites with 2 wt.% HNTs showed superior mechanical properties after six months of salty water immersion in comparison to unmodified basalt/epoxy composites. Additionally, epoxy resin modified with nanoclay (montmorillonite with dimethyl dialkyl amine) at concentrations of 0.5, 1, 1.5, 2, and 3 wt.% was used for laminating woven basalt fiber fabrics [100]. A small addition of nanoclay particle into basalt-reinforced composites was the most efficient for damping and natural frequency. The composites revealed the maximum increment of the tensile strength until 7.61%, flexural strength until 29%, and absorbed energy by impact until 16.8% at MMT concentrations of 2, 1.5, and 0.5 wt.%, accordingly. In another study, nano titanium dioxide was used for basalt fabric/epoxy composites via the vacuum-assisted resin infusion method alone and in combination with montmorillonite nano clay (at concentration of 3 wt.% TiO_2_, 3 wt.% clay or 1,5 wt.% TiO_2_, and 1,5 wt.% clay) [101]. The filled BF composite exhibited better abrasion resistance. In [102], basalt fiber-reinforced quasi-uniaxial laminate plates were fabricated by filament winding and then the vacuum bagging method. The matrix used was epoxy resin modified with 1, 2, and 3 wt.% I.30E nanocaly (primary alkylammonium ion modified Na- montmorillonite) and 1, 3, and 5 wt.% M52N (acrylic tri-block-copolymer). It should be noted that delamination fracture toughness was not affected after introducing the filler into the epoxy matrix. Nanoclay additives with their characteristic intercalated exfoliated platelets may resist crack propagation, especially by crack defection and branching; however, this effect was not observed in the case of the epoxy matrix. The formation of micelle structures after the introduction of acrylic tri-block-copolymer into epoxy resulted in cavitation during plane-strain fracture, followed by subsequent matrix deformation and toughness improvements in this epoxy system. Lowering fiber volume fraction notably increased the interlaminar fracture energy of the composites. The occurrence of interlaminar crack propagation through the composite was mostly ascribed to interfacial failure and matrix cracking.

Subagia et al. used tourmaline (TM) micro/nano particles from a group of silicate minerals (0.5–2 wt.%) with and without surfactant to modify the epoxy laminate with basalt fiber [103]. The laminates with modified epoxy matrix indicated better tensile and flexural properties than the unmodified laminates. The highest mechanical properties were found for composites containing 1 wt.% TM loading with surfactant. For this material, a 16% increase in both the tensile and flexural strength, 27.4% in tensile modulus, and 153.3% in flexural modulus were observed in comparison to pure basalt/epoxy composite. Another study [104] assessed the influence of the addition of zeolite (at concentrations of 5; 10 or 15 wt.%) and 1 wt.% POSS on the thermo-mechanical behavior of epoxy materials that were reinforced with basalt woven fabric, manufactured via hand lay-up. Silsesquioxane was applied to improve adhesion between the fiber and the matrix. Introducing zeolite and silsesquioxane to the epoxy matrix significantly increased the impact strength and thermal stability of the composites. Additionally, the dynamic mechanical thermal analysis (DMTA) confirmed that these materials were more resistant to temperature changes above the glass transition temperature. Other mineral fillers, such as basalt powder and its influence on the thermomechanical properties of basalt fabric/epoxy composites, were presented in study [105]. The application of basalt fibers with basalt powder enhanced the stiffness and thermal resistance of the epoxy composites. Moreover, the hybrid composites showed higher resistance to temperature changes than the reference sample, as confirmed by the DMTA measurements.

Surana et al. examined the effect of the addition of sawdust particles (2; 4; 6 wt.%) on the tensile and vibration properties of BF/epoxy composites [106]. Six layers of basalt fabric laminates were produced via the vacuum bagging method. The natural filler improved the tensile properties and natural frequency by 30% and 22%, respectively. However, higher filler concentration caused agglomerations of sawdust particles, which decreased the tensile and vibration properties of the laminates. Other natural fillers, such as moringa ash (at 10 wt.%) and bagasse ash (at 10 wt.%) particles, were applied to modify the epoxy matrix in basalt fiber-reinforced composites BF. Bi-directional woven basalt fiber mat was used as reinforcement [107]. The mechanical properties and chemical corrosion resistance of three types of composites were evaluated. It was found that the composites with fly ash were more resistant to chemical corrosion than the reference sample. The tensile strength of the composites with moringa ash had higher ultimate strength (138.6 MPa), 17% higher than the composite with bagasse ash (113.39 MPa), and 47% of the reference composite (84.7 MPa). This phenomenon was due to the presence of the ash particle, which enhanced the matrix yield strength. Moreover, silica particles that were present in the moringa ash improved the impact strength of the moringa-modified composite in comparison to the bagasse-modified composite and unmodified composites. 

A natural origin basalt fiber is more environmentally friendly than other reinforcing fibers. Additionally used in composites, it allows for obtaining similar results to materials that were reinforced with carbon fiber. Therefore, the largest number of studies referred to in this review concerned the use of basalt fibers in hybrid composites. In most cases, vacuum assisted and hot pressing techniques were used to produce this group of materials. The characteristics of basalt fiber reinforced hybrid composites are presented in Table 3. Also in their manufacture, carbon modifiers such as CNTs, GNPs, NGF and GO are used in many cases. For nanomodifiers, concentrations up to 1 wt.% were used, while graphite flake powder was introduced in amounts up to 40 wt.%. When CNTs were used as an epoxy resin modifier, its surface modification in the salinization process proved beneficial and resulted in improvement of mechanical and thermal properties of composites. Additionally, surface modification of graphene nanoplatelets with silane improved the adhesion between the fibers and the matrix, which increased the microhardness and wear properties of composites. On the other hand, graphite flakes significantly increase the mechanical characteristics of basalt-reinforced composites when incorporated into the epoxy matrix at a concentration of up to 20 wt.%. Halloysite nanotubes applied in a concentration up to 2 wt.% also had a positive effect on strengthening and stiffening the basalt fiber/epoxy composite structure. An interesting solution was the use of both MMT and nano titanium dioxide as the epoxy matrix modifier, which significantly improved the wear resistance of the composites. Likewise, the modification of the epoxy matrix with mineral fillers, such as tourmaline, zeolite, or basalt powder, leads to an improvement in the thermomechanical properties of basalt fiber reinforced composites. The number of studies on this subject is constantly growing, providing important information on the possibilities of their industrial application, due to the attractive properties that result from the use of basalt fibers in polymer composites.

## 3. Conclusions

This review presents various studies into the mechanical and thermo mechanical properties of hybrid epoxy composites with both powder and fiber fillers. In particular, composites that were reinforced with glass, carbon, and basalt fabrics obtained by various lamination techniques are characterized. Laminates are composed mainly of: fibers that are responsible for transferring the load to the composite, a matrix that provides the volume of the composite and transfers the load between the fibers, and a fiber-matrix interface that should provide adequate interphase strength of the composite. Therefore, selecting the accurate method of epoxy matrix modification requires the consideration of many factors that are related to the structure of individual components and the way they are processed. Most literature reports concern the use of nano- or micro-powder fillers, such as carbon nanotubes, graphene derivatives, nanoclays, and silica, to modify the epoxy matrix. Using the simultaneous modification of the epoxy matrix with powder filler and reinforcing fiber, beneficial improvement of such properties as the tensile and flexural strength, interlaminar shear strength, impact strength, fracture toughness, ballistic impact behavior, viscoelastic, and tribological were obtained. In the case of nanofillers, the typically applied percentages of additives did not exceed 1 wt.%, while for other microfillers they were significantly higher. In most cases, the use of carbon nanotubes introduced into the resin (up to 1 wt.%) improved the mechanical properties of composites and the adhesion between the fiber and the matrix. Favorable results were also noted for hybrid materials that were modified with graphene nanoplatelets (up to 2 wt.%). On the other hand, materials modified with montmorillonite in an amount of up to 5 wt.% showed superior properties. In addition, for carbon fiber composites, the introduction of nano-SiO_2_ into epoxy resin (up to 8 wt.%) contributed to improving the properties of these laminates. The epoxy matrix should be modified in such a way as to ensure good adhesion between all components in the laminate in order to provide increased mechanical and thermomechanical properties of hybrid composites. It should be emphasized that for all these types of analyzed hybrid composites reinforced with glass, carbon and basalt fibers, respectively, carbon-based nanomodifiers proved to be the most effective powder filler. Their small addition significantly improved the adhesion of the fiber to the matrix, which translated into the observed effect of strengthening and improving their thermal and mechanical properties.

## Figures and Tables

**Table 1:** Characteristics of glass fiber reinforced hybrid composites.

Matrix	Type of Fiber	Powder Filler	Powder Filler wt.%	Filler Features	Mixing Method	Fiber Layers,	Technology	Improved Properties	Ref.
Epoxy/cycloaliphatic amine	E-glass woven fabric with 0.5 wt.% epoxy silanes	amino-MWCNTs	0.1–0.4	diameter 10 nm	manually mixed and sonication with epoxy monomer	7	hand lay-up compression hot press techniques	flexural property, thermal stability, storage modulus glass transition temperature	[51]
Epoxy/ tetraethlyenepentamine and nonylphenol	bidirectional E-glass woven fabric	nanoclay Cloisite 30B	2; 4	1 nm thickness 200–300 nm in length	hot plate/magnetic stirring with curing agent	6	hand lay-up compression technique	the ultimate tensile strength, tensile modulus, tensile ductility fatigue strength fatigue life	[52]
Epoxy/amine	unidirectional E-glass fabric	GNPs	2	surface area 500 m^2^/g	mechanical stirring with epoxy monomer	4	hand lay-up	flexural and tensile properties after post-curing process	[53]
Epoxy/aromatic amine	unidirectional E-glass fabric	amino GNPs	12	surface area: 500–700 m^2^/g	sonication with epoxy monomer	50%	hand lay-up compression hot press techniques	was not observed	[54]
Epoxy/amine	unidirectional E-glass fabric	GNPs	0.1; 0.3; 0.5	outer diameter and thickness of 10–20 nm	ultrasonic mixing with epoxy system	9	hand lay-up compression hot press techniques	tensile strength	[55]
Epoxy/amine	glass woven fabric	MMT: Cloisite 93	1; 3; 5; 7	<40 µm	mechanical ultra-sonicator with epoxy	–	hand lay-up compression hot press techniques	tensile strength flexural strength impact strength	[59]
Epoxy/amine	glass woven fabric	CMCs	1; 1.5; 3	~50 μm particle size	sonication with epoxy monomer	6	vacuum infusion process	ILSS, tensile strengths, flexural strengths, storage modulus glass transition temperature	[60]
Epoxy/amine	bidirectional glass woven fabric	g-C_3_N_4_	1; 1.5; 2; 2.5; 3	300–600 nm particle size, surface area 9.4 m^2^/g	mechanical stirring with epoxy system	12	hand lay-up compression technique	ILSS, tensile strength, flexural strength	[61]
Epoxy/ amine	woven E-glass fiber	bamboo powder	10; 20; 30	500 μm–1 mm particle size	mechanical stirring with epoxy system	2	hand lay-up method compression moulding technique	tensile strength flexural strength	[64]

**Table 2:** Characteristics of carbon fiber reinforced hybrid composites.

Matrix	Type of Fiber	Powder Filler	Powder Filler wt.%	Filler Features	Mixing Method	Fiber Layers	Technology	Improved Properties	Ref.
Epoxy/ polyamine	unidirectional carbon fabric	MWCNTs GNPs	0.5; 1 0.5; 1	outer diameter 10–15 nm, length >500 nm; surface area 100 m^2^/g	high shear mixing with epoxy monomer	14	hand lay-up	interlaminar fracture toughness	[71]
Epoxy/amine	satin weave carbon fabric	CNTs amino- CNTs	0.1, 0.2, 0.3 0.1, 0.2, 0.3	diameter 9.5 nm length <1 μm, diameter 9.5 nm length <1 μm	calendering by laboratory scale three roll mill	8	hot mixing VARIM	flexural strength, intra and interlaminar strengths	[72]
Epoxy/amine	carbon fabric	MWCNTs SWCNTs	0.25 0.25	length of ~2–6 µm	carbon fabric was soaked in the CNTs solution	4	VARTM	ILSS, out-of-plane electrical conductivity	[73]
Epoxy/anidrydic	winding continuous carbon fibers	MMTs:			high speed mixing with hardener	50% vol.	compression hot press techniques	tensile properties, energy absorption capability	[76]
		Cloisite 30B	2; 5	2–13 µm					
		Cloisite 25A	2; 5	2–13 µm					
		Cloisite 15A	2; 5	2–13 µm					
Epoxy/amine	unidirectional carbon fabric	MMTs	2; 4; 6; 8	cation exch. cap. Na-MMT 79 mmol/ 100 g	mechanically stirred	10	hand lay-up and compression molding	ILSS, flexural strength	[78]
Epoxy/polyamine	carbon fabric	nano-SiO_2_	1; 2; 3; 4; 5; 6; 7; 8	average diameter of 20 nm	mechanically stirred with epoxy system	4	vacuum infusion method	impact properties	[80]
Epoxy/amine	carbon/Kevlar twill fiber fabric	nano-SiO_2_	0.5; 1; 1.5; 2.5; 3	diameter 1–10 nm width of 0.5–2 μm	manually mixed with epoxy system	8	hand lay-up	tensile strength, flexural strength	[83]

**Table 3:** Characteristics of basalt fiber reinforced hybrid composites.

Matrix	Type of Fiber	Powder Filler	Powder Filler wt.%	Filler Features	Mixing Method	Fiber Layers	Technology	Improved Properties	Ref.
Epoxy/ polyamidoamine	woven-type basalt fibers	silanized CNTs	1	diameter 10–15 nm length 10–20 nm	mechanical stirring with epoxy system	8	hand lay-up autoclave method	vibration damping coefficient	[89]
Epoxy/ polyamidoamine	woven-type basalt fiber	CNTs oxidized CNTs silanized CNTs	1 1 1	diameter 10–15 nm length 10–20 nm	mechanical stirring with epoxy system	4	VARTM autoclave method	tensile properties, thermal stability, storage modulus	[90]
Epoxy/ polyamidoamine	woven-type basalt fiber	CNTs: oxidized, silanized	1 1 1	diameter 10–15 nm length 10–20 nm	mechanical stirring with epoxy system	8	VARTM autoclave method	flexural modulus, flexural strength	[91]
Epoxy/amine	basalt plain fabric	GNPs	0.1; 0.2; 0.3	diameter 5 µm surface area: 150 m^2^/g	mechanical stirring with epoxy system	10	hand lay-up	tensile strength, flexural strength, impact resistance	[92]
Epoxy/amine	woven S-glass fabrics; basalt fabric	GNPs	0.1;0.25; 0.5	diameter 5 µm thickness 5-8 nm surface area 150 m^2^/g	mechanical stirring with epoxy system	12	hand lay-up and VARTM	impact properties, mechanical properties	[93]
Epoxy/polyamine	woven basalt fibers	silanized GNPs	0.1; 0.2; 0.3; 0.4; 0.5	diameter 12–18 µm	mechanical stirring and ultrasonicated with epoxy system	12	hand lay-up compression hot press techniques	microhardness, wear properties	[94]
Epoxy/DDM	woven-type basalt fibers	graphite flake powder	10; 20; 30; 40	12.01 g/mol, particle size	mixed in a planetary mixer		hand lay-up compression hot press techniques	mechanical properties: critical stress intensity factor; critical strain energy release rate	[95]
Epoxy/amine	basalt roving fibers	silane-treated GO	0.1; 0.2; 0.3; 0.4; 0.5	diameter 10-50µm; thickness of 3.4–7 nm	sonication with epoxy monomer	6 24	hand lay-up technique under static compression	tensile strength, flexural strength compressive strength	[96]
Epoxy/amine	woven fabric basalt fiber	HNT	1; 2; 3	diameter 20–40 nm	sonication with epoxy monomer	12	vacuum assisted resin infusion	mechanical properties	[99]
Epoxy/amine	plain and woven basalt fiber fabrics	MMT	0.5;1; 1.5; 2; 3	lateral width 0.5–2 μm, thickness 1–10 nm	mechanical stirring with epoxy system	12	VARTM	tensile strength, flexural strength, absorbed energy by impact	[100]
Epoxy/aliphaticamine	woven fabric basalt fiber	Nano-TiO_2_ MMT TiO_2_ + MMT	3 3 1.5 + 1.5	particle size: 25 nm surface area 220–270 m^2^/g	mechanical stirring with epoxy system	3	vacuum assisted resin infusion	abrasion resistance	[101]
Epoxy/amine	woven fabric basalt fiber	tourmaline powder	0.5; 1; 2	particle size: 900 nm–8 μm	mechanical stirring with epoxy system	10	VARTM	tensile strength, flexural strength	[103]
Epoxy/amine	woven fabric basalt fiber	zeolite POSS	2.5; 5; 10; 1	particle size: 20 μm	mechanical stirring with epoxy system	10	hand lay-up	impact strength, thermomechanical properties, thermal stability	[104]
Epoxy/amine	woven fabric basalt fiber	basalt powder	2.5; 5; 10	particle size: ~10 μm	mechanical stirring with epoxy system	6	hand lay-up	stiffness, thermal resistance	[105]
